# Deployment of a Smart Structural Health Monitoring System for Long-Span Arch Bridges: A Review and a Case Study

**DOI:** 10.3390/s17092151

**Published:** 2017-09-19

**Authors:** Zengshun Chen, Xiao Zhou, Xu Wang, Lili Dong, Yuanhao Qian

**Affiliations:** 1 State Key Laboratory Breeding Base of Mountain Bridge and Tunnel Engineering, Chongqing Jiaotong University, Chongqing 400074, China; zchenba@connect.ust.hk (Z.C.); zhouxiaocqjtu@gmail.com (X.Z.); armelejos@hotmail.com (Y.Q.); 2 Department of Civil and Environmental Engineering, The Hong Kong University of Science and Technology, Hong Kong, China; 3 The Key Laboratory for Health Monitoring and Control of Large Structures, Shijiazhuang 050043, China; 4 Department of Civil and Environmental Engineering, University of Illinois Urbana-Champaign, Urbana, IL 61801, USA

**Keywords:** structural health monitoring, smart system, long-span arch bridge, intelligent management

## Abstract

Structural health monitoring (SHM) technology for surveillance and evaluation of existing and newly built long-span bridges has been widely developed, and the significance of the technique has been recognized by many administrative authorities. The paper reviews the recent progress of the SHM technology that has been applied to long-span bridges. The deployment of a SHM system is introduced. Subsequently, the data analysis and condition assessment including techniques on modal identification, methods on signal processing, and damage identification were reviewed and summarized. A case study about a SHM system of a long-span arch bridge (the Jiubao bridge in China) was systematically incorporated in each part to advance our understanding of deployment and investigation of a SHM system for long-span arch bridges. The applications of SHM systems of long-span arch bridge were also introduced. From the illustrations, the challenges and future trends for development a SHM system were concluded.

## 1. Introduction

With the development of economy and society, many long-span bridges have been built or are under construction all over the world, i.e., the Akashi Kaikyo Bridge (suspension bridge, main span 1991 m) in Japan, the Sutong Bridge (main span 1088 m) in China, the Chaotianmen Bridge (arch bridge, main span 552 m) in China [1,2]. The long-span bridges should meet the requirement of the serviceability, safety and sustainability during the operation stage. However, global climate change tends to present many challenges for long-span bridges such as stronger hurricanes and faster material deterioration. A considerable number of long-span bridges were destroyed due to natural and man-made hazards. For example, the famous Tacoma Narrow Bridge collapsed due to the strong wind [3], the I-35W Bridge in USA collapsed in 2007 was initiated by buckling at the portions connecting the diagonal members under compressive axial loads, and the Wenchuan earthquake happened in China in 2008 leaded to the collapse of many bridges [4]. Due to the appealing appearance and the good mechanical properties, many long-span arch bridges have also been widely built worldwide. The deterioration of main arch rings, arch column, tie rod or hanger rod of an arch bridge is mainly caused by vehicle load, environmental hazard, man-made hazard and their coupled effects. The serviceability, safety and sustainability of the long-span bridges including arch bridges have received much attention by administrative authorities. A structural health monitoring (SHM) system that is used for surveillance, evaluation and assessment of the condition of existing long-span bridges has been widely developed, and the recently-developed long-term SHM system is one of cutting-edge systems for monitoring the serviceability, safety, and sustainability of long-span bridges [5]. 

SHM systems take advantage of new sensing technologies that have been developed over the past two decades. Fiber Optic Sensors (FOP) and wireless techniques have been widely used because of the advantages and the huge benefits for applications characterized by a difficult access to the structure [6,7]. The miniaturization of sensors, represented by the so-called Micro Electro-Mechanical Systems (MEMS) has also received much attention [8,9]. The company, Intelligent Sensing for Innovative Structures (ISIS, Canada), has equipped up to six bridges with fiber optic sensing systems that allow remote monitoring since 1993 [10]. The Siggenthal Bridge in Switzerland which is an arch bridge with the main span of 117 m has installed 58 FOPs [11]. Other bridges, such as the Ebian Dadu river arch bridge in China [12], the Flexi-Arch in United Kingdom [13], the Versoix Bridge in Switzerland [14] and the Yonghe Bridge in China [15], adopted the FOP for sensing. The studies and applications have pointed out that FOP sensor has a long sensing range and the capability of providing strain or temperature at every spatial resolution along the entire sensing fiber, imbedded in or attached to the structures, using the fiber itself as the sensing medium, and have affirmed that FOP sensors are more accurate and reliable than other sensors (i.e., strain gauge) [16,17,18,19]. Wireless sensors have also been widely employed and installed in SHMs of bridges [20,21,22]. All the investigations and applications of wireless sensors have indicated that it is potential to employ the wireless technique in bridge monitoring and management. 

Taking advantage of the new sensing techniques, long-term SHM systems for long-span bridges have been developed. A SHM system has been implemented in the Lupu bridge which is a steel half-trough tied arch bridge and the second longest arch bridge in the world. The temperature, strain, acceleration, and wind effect of the bridge were monitored by the system [23]. Alamdari et al. [24] have presented a large scale SHM application for the Sydney Harbour Bridge which is an arch bridge with a main span of 503 m. The performance and structural damages of a subset of 800 jack arches under the traffic lane 7 were analyzed based on the data acquired from the SHM system. Magalhaes et al. [25] have developed a SHM system for the Infante D. Henrique Bridge in Portugal which is a concrete arch bridge with a main span of 280 m, to evaluate the usefulness of approaches based on modal parameters tracking for SHM of bridges. Ding et al. [26] have investigated dynamic characteristics of the hanger vibration of a high-speed railway arch bridge, the Dashengguan Yangtse River Bridge in China, by using the data observed from a SHM system. Apart from the above SHM systems, many other SHM systems have been installed in long-span bridges [27,28,29]. Typical examples include the Sutong bridge (1088 m, a cable-stayed bridge in China) [30,31], the Tsingma bridge (1337 m, a suspension bridge in Hong Kong) [32], the Tatara Bridge (890 m, a cable-stayed bridge in Japan), the Akashi Kaikyo Bridge (1991 m, a suspension bridge in Japan) [33], the Great Belt East Bridge (1624 m, a suspension bridge in Denmark) [34], the Normandie Bridge (856 m, a cable-stayed bridge in France) [35], the Commodore Barry Bridge (548 m, a truss bridge in USA) [36], and the Confederation Bridge (250 m, a box girder bridge in Canada) [37]. In Hong Kong and mainland China alone, more than 80 bridges had been equipped with SHM systems by the year of 2016 [17]. These SHM systems advance our understanding of development of a long-term system. More importantly, the data observed from the SHM systems can be utilized for evaluating the serviceability, safety, and sustainability of long-span bridges.

Many researches have made the efforts to the parameter identification, damage detection, model updating, safety evaluation and sustainability assessment of long-span bridges by using the data observed from SHM systems. Rainieri and Fabbrocino [28,38,39,40,41] have identified modal parameters of civil structures including arch structures based on the data observed from SHM systems by using modal-based damage detection algorithms. Kurt et al. [42] proposed a new nonlinear model updating strategy based on global or local nonlinear system identifications. The reduced-order models of a dynamical system have been proposed and updated based on SHM observed data. Comanducci et al. [43] have presented applications of different vibration-based damage detection methods by using up-to-date multivariate statistical analysis techniques based on the data observed from a SHM system of a long-span arch bridge. The results focusing on the assessment of the minimum detectable damage severity using different techniques are anticipated to contribute a more aware use of monitoring data and reliance over related health state assessment information. Li et al. [44] have investigated damage identification of a streamline bridge model by using SHM observed data. Li and Ou [45] and Li et al. [46] have summarized bridge health diagnosis based on SHM systems. 

The development of modern information and communication system, signal processing technology, internet and structural analysis significantly advances the application and improvement of the SHM systems. Despite these advancements, there still exist big challenges in SHM, which need to be addressed in the future, such as the improvement of the accuracy of a sensory system, high-frequency and accurate data sampling, data mining and knowledge discovery, diagnostic methods, the analyzing and modeling the bigdata observed from the SHM system utilized for decision making on maintenance and management [6,31,47,48,49,50,51,52]. This study explores the recent progress of the SHM of long-span arch bridges. Section 1 briefly introduces the background of SHM systems for long-span arch bridges. Section 2 reviews the deployment of SHM systems for long-span arch bridges. Section 3 summarizes the approaches for data analysis, modeling and safety evaluation of long-span arch bridges. Based on Section 2 and Section 3, Section 4 summarizes the challenges and future trends in SHM systems for long-span arch bridges. Section 5 summarizes the application of SHM systems in arch bridges. It should be noted that a case study about SHM of a long-span arch bridge (Jiubao Bridge in China) is incorporated in each section for explanation. Section 6 concludes the main contribution of the present study.

## 2. Deployment of SHM Systems for Long-Span Arch Bridges

### 2.1. Overview of a SHM System

**Figure 1:**
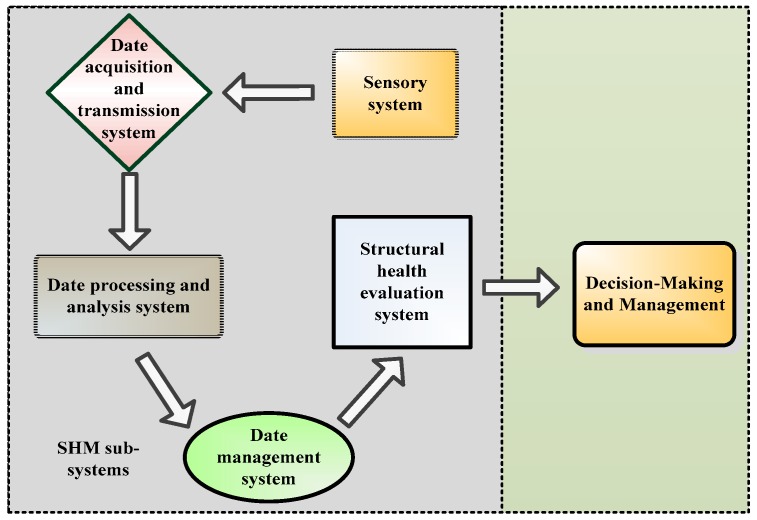
Subsystems of a long-term SHM system.

As mentioned before, the main goal of a SHM system is to the serviceability, safety, and sustainability of long-span arch bridges. To achieve the goal, a long-term SHM system should include at least five integrated sub-systems [30,53], as shown in Figure 1. 

In Figure 1, the function of each sub-system is briefly summarized as: (1) the sensory sub-system is utilized for sensing the information of the working environment of a bridge and various factors that affect the safety of the bridge, such as wind speed and wind direction, environmental temperature and humidity monitoring, bridge load vehicle, vibration, structural temperature, strain, main beam linear, support displacement and cable tension; (2) the data acquisition and transmission subsystem is used to sample and transmit the information sensed by the sensory subsystem; (3) the data processing and analysis subsystem is used to process and analyze the obtained data so that the data can be conveniently utilized for further analysis; (4) the data management subsystem is used to receive and storage the observed data; (4) the structural health evaluation subsystem is utilized to evaluate and assess the condition of a long-span arch bridge; (5) the decision-making and management sub-system reflects the condition of the monitored objective, which is easily utilized by bridge manager to make decisions about the objective, such as maintenance, repair, reinforcement and re-built of the objective. A SHM system including the subsystems is envisaged to (1) obtain numerous situ data that can be utilized for leading-edge research; (2) provide real-time information for safety assessment; (3) provide information for prioritizing bridge maintenance and repair; (4) detect anomalies in loading, response, deterioration and damage to ensure structural operation safety; (5) validate design assumptions and parameters that are benefit for improving design specifications and guidelines [54]. The deployment of each sub-system is reviewed below.

### 2.2. Sensory Sub-System

As pointed out in a previous study [49], there are mainly three aspects considered in a sensory sub-system: the variable type, the sensor type, and the positioning of the installation of sensors. The variables are categorized into load and environmental actions, global response, and local response. Specifically, the load and environmental actions mainly include vehicle load, wind load, earthquake ground motion, vessel collision, temperature, humidity, etc. The global response mainly includes acceleration, deformation whereas the local response includes strain, cable tension force (hanger), displacements of joints and bearings, crack and fatigue of elements, as well as responses of piers. Based on the variables in a SHM system, sensors should be installed to be able to measure: vehicle loads, wind speeds & direction, environmental temperature & humidity, vibration, structural temperature, strain, main beam deflection, bearing displacement, and cable tension force. 

Vehicle loads including traffic flow, vehicle speed and vehicle weight of each axle, numbers of axles are often acquired by a weigh-in-motion (WIM) system [55,56]. A WIM system should be installed in all lanes of a cross section of an arch bridge. When a vehicle passes through the bridge, it provides a reference for the establishment of the bridge traffic load model and the evaluation of the bridge.

Aerodynamics of a long-span bridge are complicated due to the complex wind-induced vibrations in cross-wind direction, i.e., buffeting, vortex shedding, flutter and the combined interaction [57,58,59]. Mechanical and ultrasonic anemometers are often applied to monitor the variation of wind speed and wind direction at a bridge site. The data collected at the site can be used to identify wind characteristics (e.g., mean wind speed, prevalent wind direction, turbulence intensity, and wind power spectrum) and to evaluate the effect of wind on a bridge. Compared with mechanical anemometers, ultrasonic anemometers have the characteristics of high-precision, high resolution, durability, long life-span and maintenance free; However, their disadvantage is that the range is relatively small.

The effect of earthquake on piers of a bridge can be obtained by using three-direction seismometers which should be installed on piles of bridge piers or installed at the free field far away from the bridge. The vessel collision can also be obtained by using accelerometer or seismometer installed on the piles of bridge piers.

It should be noted that the main load-carrying member of an arch bridge is the arch ring, the vibration of which should be well monitored. The vibration as well as the dynamic characteristics of the arch and the main girder in real-time, can be recorded by using acceleration sensors. The sensors record the major loads and accidents that the arch and the main girder experience. The acquired data provides the data support for the damage evaluation of the bridge. The displacement can be observed by pressure transmitter sensor. 

Strain is one of the most important variables and it directly reflects the condition of a monitoring bridge. The measured data can be utilized for safety and sustainability evaluation, and fatigue assessment. There are many types of strain gauge, such as a traditional strain gauge, a vibrating-wire strain gauge and optical fiber Bragg grating (FPG) strain sensors. Due to the advantages of the FPG stain gauge mentioned before, FPG stain sensors are widely used by a SHM system [60]. The Boguan Bridge, Yingzhou Bridge, Xinguang Bridge, Tongshunlu Bridge, and Ebian Dadu Bridge, which are long-span arch bridges in China [17], embedded FPG sensors into SHM systems. The strain sensors can be well distributed according to a structural analysis and fragility analysis. 

For tied-arch bridges, the hanger rod/cable is the main member of the bridges, and the forces of hanger rod/cable should be well monitored. The monitoring variables mainly include vibration, tension force, fatigue damage, and corrosion. Acceleration sensors with Lens testing are often used to monitor suspender cable force and wind-rain induced vibration, and fiber brag grating test-force rings are used to monitor the force of short suspender, cable hanger and outside of prestressed cables [15,31,61].

The sensors often utilized in a sensory sub-system are summarized in Table 1.

### 2.3. Data Acquisition and Transmission Sub-System

**Figure 2:**
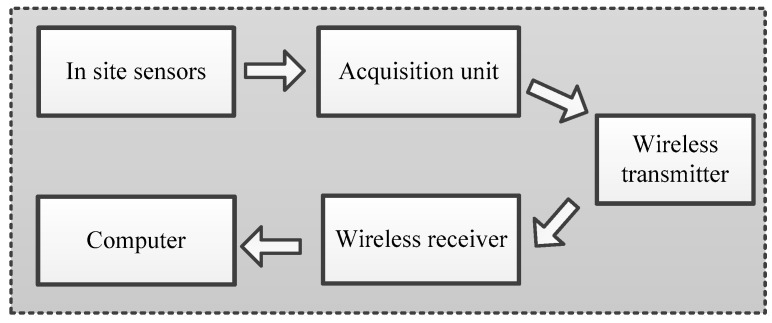
A framework of data acquisition and transmission sub-system.

The data acquisition and transmission sub-system mainly includes selecting data acquisition devices and method, and sampling modes, as well as the transmission technology. Paek and Caffrey [62] have introduced the deployment experiences and evaluated the performance of a multi-hop wireless data acquisition system (called Wisden) for SHM of structures. The system has been validated by employing the system to a large structure. Ni et al. [63] have introduced a wireless system that is utilized for synchronous acquisition of strain and temperature data and real-time data transmission from substations to office. It consists of three parts: WiFi router, wireless bridge, and antenna. Li and Ou [49,64] have introduced the details of design approaches of a data acquisition and transmission systems. From the above studies, a framework for data acquisition and transmission sub-system is briefly summarized in Figure 2. It is not hard to design this sub-system. However, there still exists problems for high-frequency and accurate acquisition and transmission. The sampling rate for dynamic signal should follow the Nyquist Shannon sampling theorem. Related sampling theory can refer previous studies [65,66]. Additionally, the signal distortion caused by long-distance transmission should also be well concerned [67]. 

### 2.4. Data Processing, Management, and User Interface

The data processing and management system can process all dynamic and static data during the whole life cycle of a bridge, including the query, storage, etc. of the data. It also releases information to user interface. Based on the equipment in the monitoring center, remote monitor of full-bridge can be achieved. The center is mainly equipped with server group, central network switching equipment, server maintenance equipment, optic fiber grating strain acquisition station, workstations and other equipment.

The structural health evaluation subsystem can evaluate the safety of bridge according to the information on the strain, deformation, temperature and cable force signals at the key parts of the bridge. Corresponding analysis and evaluation methods will be introduced in Section 3. According to monitoring data, the identification of structural state and damage can be completed. Intelligent early warning analysis platform can provide alarm monitoring scheme in real-time, which send monitoring report on a daily basis. Once an early warning signal is generated, the alert would be released immediately. 

The user interface of off-line data analysis is developed by proper software programs, i.e., MATLAB and .NET platform, VB, C++. It consists of several modules for the relative of parameters, nonlinear regression, cable force, vibration, etc. Through the user interface, the condition of a bridge can be easily monitored. 

### 2.5. Deployment of SHM for a Long-Span Arch Bridge

#### 2.5.1. Description of an Arch Bridge 

**Figure 3:**
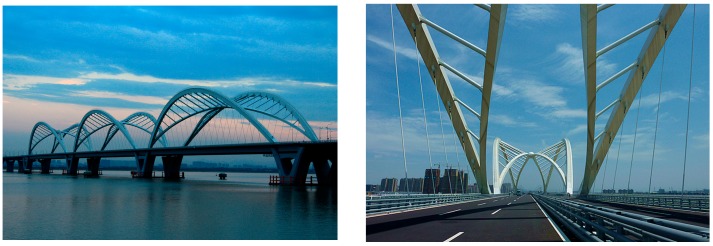
Overview of the Jiubao Bridge.

To illustrate the deployment of a SHM system for an arch bridge, the Jiubao Bridge is taken as a case study. The Jiubao Bridge is a tied-arch bridge and it has been built in 2012 in Hangzhou, China. The overall length of the bridge is 1855 m with the span arrangement of 55 m +2 × 85 m +90 m (north approach span) +3 × 210 m (main navigation span) + 90 m +9 × 85 + 55 m (south approach span). The main navigational span with an arrangement of 3 × 210 m was constructed by the continuous hybrid arch-girder superstructure combined with a steel-beam arch composite system. The bridge deck is composed of open steel boxes and concrete slabs (in Figure 3). 

The substructure of the main bridge is made of V-shape and thin-wall piers. The superstructure of the main bridge is of steel birder composite beams, which is continuous with arrangement of 188 m +22 m +188 m +22 m +188 m. The substructure of the approach bridge is made of single-plant and hollow piers, and the pile cap adopts chamfer rectangle form. The pile foundation adopts five 1.8 m diameter cast-in-situ concrete piles with the length of 90–95 m. The approach bridge is 85 m as standard span, of which the superstructure is design as single box and single chamber section of large cantilever and constant height, and of continuous box beam of steel-concrete composite girder bridge. 

To acquire the behaviors and performance of real, full-scale bridges under real loadings and environmental conditions and to further ensure the safety, serviceability, durability, and sustainability of the bridge, a smart SHM system that consists of automatic data acquisition and monitoring subsystem, data management and processing subsystem, monitoring and evaluation subsystem, comprehensive pre-alarming and safety evaluation subsystem has been implemented to the bridge. 

#### 2.5.2. Overview of the SHM System

The entire system consists of four main components, including automatic data acquisition and monitoring subsystem, data management and processing subsystem, monitoring and evaluation subsystem, comprehensive pre-alarming, and safety evaluation subsystem. Sensors are used to first convert the collected data into electrical (light) signal. The data acquisition units deployed in the main box girder then allow to upload the data to the collection station in the middle and lower reaches of main arch. Finally, the data is transferred to the monitoring center. By providing effective information sources or mechanical indexes to other subsystems, the program is set according to the demands, which could achieve intelligent control monitoring parameters of the collection. Based on the monitoring data, the technical state and bearing capacity assessment was evaluated, which provide a scientific basis for maintenance. 

**Figure 4:**
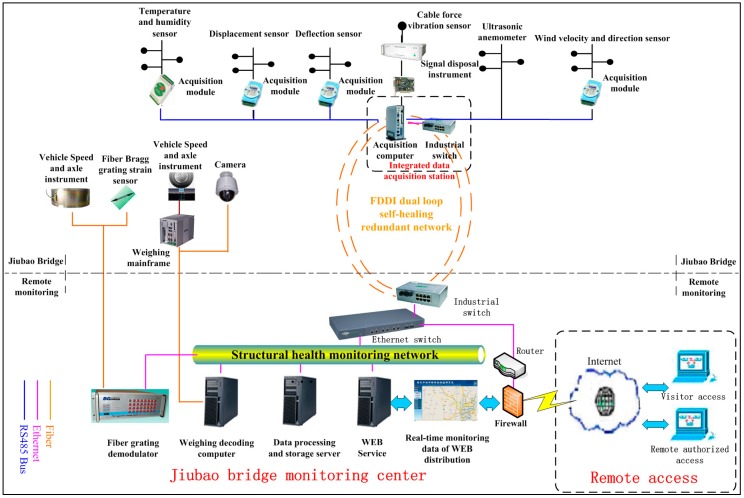
Flowchart of data acquisition and transmission of automatic monitoring system.

The data acquisition and transmission scheme of the automatic monitoring system is composed of a data acquisition station in the external field, a server group in monitoring center and a fiber optical signal transmission network. Field data acquisition station consists of several data acquisition modules, and the modules can ensure the stability, durability and high precision of the system by using advanced and mature products. In order to ensure the reliability of signal transmission, FDDI double loop topology is used as optical fiber signal transmission network. Details of the data acquisition and transmission of automatic monitoring system are presented in Figure 4.

#### 2.5.3. Sensory Sub-System

**Figure 5:**
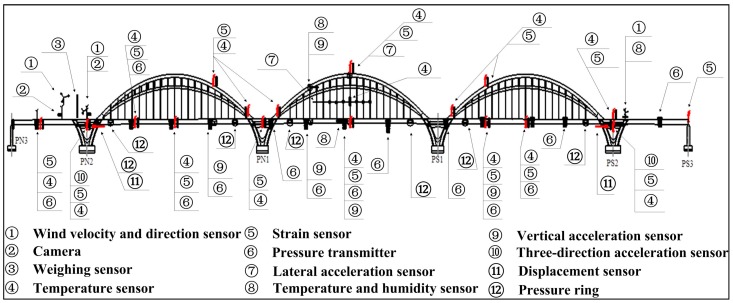

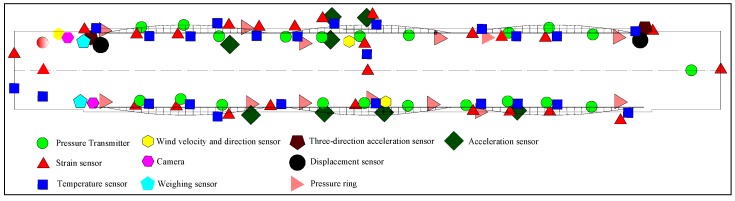
Layout of sensors on Jiubao Bridge.

Sensors are installed to the bridge to obtain wind speeds & direction, environmental temperature & humidity, vehicle loads, vibration, structural temperature, strain, main beam deflection, bearing displacement, and cable tension force. Eleven types of sensors were mounted on the bridge, including wind velocity and direction sensors, temperature and humidity sensors, acceleration sensors, speed and axle meters, digital camera, temperature sensors, pressure transmitter, displacement sensors, strain sensors, pressure ring, and vibration sensors. There are 333 sensors in total installed on the bridge. Sensors installed in the arch bridge are listed in Table 1. Figure 5 shows the layout of sensors installed on the main bridge.

#### 2.5.4. Data Acquisition and Transmission Sub-System

The main optical fiber transmission network includes FDDI double ring optical Ethernet transmission network and field bus transmission network. FDDI double ring optical Ethernet transmission network consists of field integrated data acquisition station and server group of monitoring center through the optical fiber link. Field data acquisition station links acquisition module or sensor communicate to bus transmission network. To avoid signal distortion caused by long-distance transmission, the method of extensive distribution of the system sensors, centralized control, distributed acquisition, local storage and data uploading pattern are employed in the field data acquisition station, which includes collection host and several acquisition modules (Figure 4). The acquisition module and the acquisition host are connected to RS485 bus. The various modules can collect different signals such as voltage, current and temperature and humidity signals corresponding to wind velocity and wind direction, alignment, deviation of tower, displacement, temperature and humidity and other parameters. The length of RS485 bus connection is about 1200 meters and can be increased by the RS485 repeaters. 

The signal collected from vibration sensors is firstly transferred to signal conditioning by electric cable, and then is transferred to the PCI dynamic data acquisition card for collection. Acquisition computer and dynamic data card use PCI bus to perform the data acquisition, transmission and storage.

**Figure 6:**
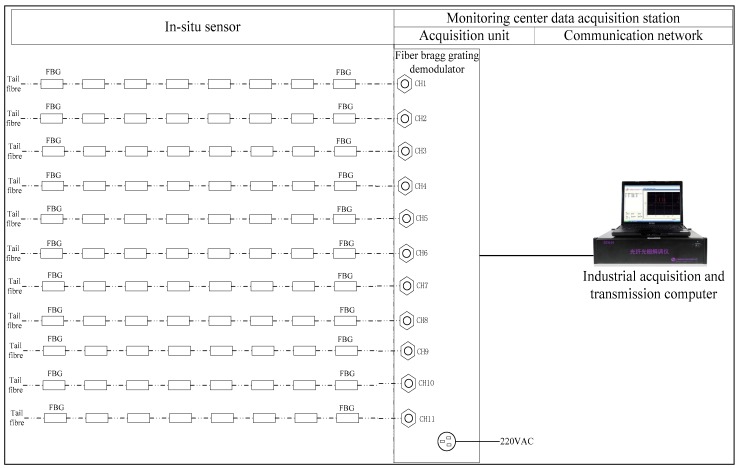
Framework of data acquisition and transmission network topology for stress-strain and cable force monitoring.

After receiving the reflected light by FBG demodulator, the calculation of wave length can be completed. And then the data from calculation will be stored and at the same time upload to the data base in monitoring center. The network topology diagram is shown in Figure 6.

**Figure 7:**
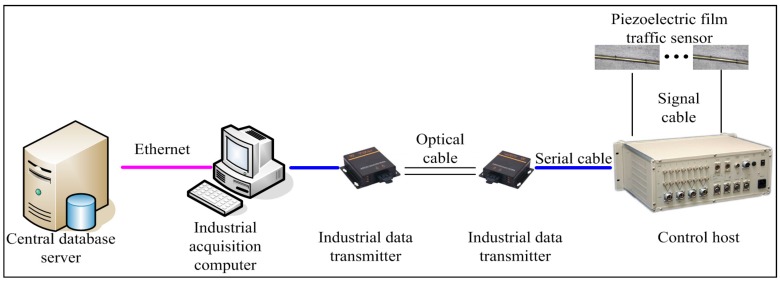
Flowchart of data acquisition and transmission network topology for traffic flow.

Traffic flow monitoring system is responsible for weigh in motion (WIM), velocity measurement and statistics of vehicle flow. Traffic flow monitoring system terminal communicates by computers in monitoring center and fiber ethernet, and the monitoring data store in local data base and upload to server in preparation for invoking and querying. The network topology diagram of traffic flow monitoring system is shown in Figure 7.

#### 2.5.5. Data Processing, Management, and User Interface

**Figure 8:**
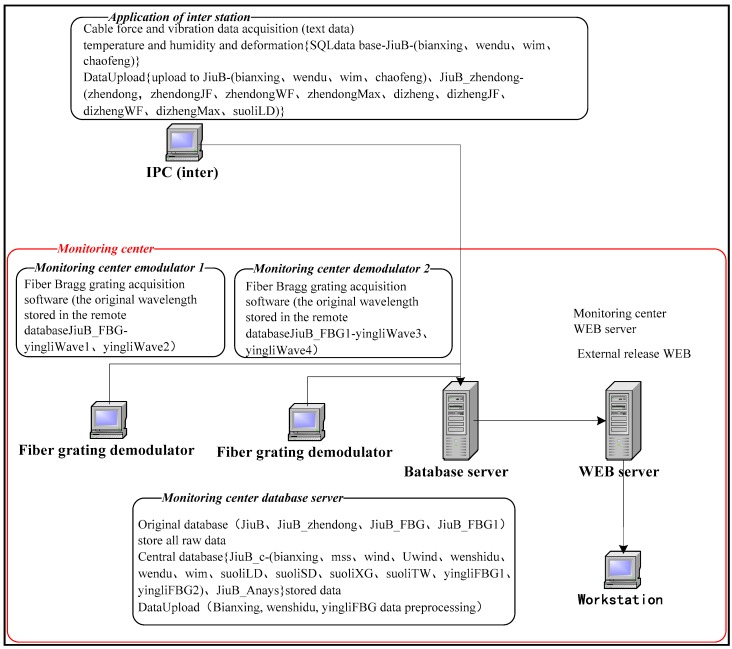
Diagram of equipment function in monitoring center.

The industrial acquisition PC located in the steel box girder of the bridge connected with the monitoring center device by the trunk optical cable, and data exchange is fulfilled by the switches installed in the monitoring center. The function and topology structure of the devices in monitoring center are shown in Figure 8.

## 3. Data Analysis and Condition Evaluation

### 3.1. Techniques on Modal Identification

By using the response observed from a SHM system, dynamic parameters of a structure can be obtained. The conventional methods used for modal identification include the peak-picking (PP) method, the random decrement technique (RDT), the frequency domain decomposition (FDD) method, as well as some time-domain covariance-drive methods reported in a previous study [68]. The PP, RDT and FDD methods ae well as their combinations that are often utilized for modal identification are introduced below. 

#### 3.1.1. The Peak-Picking (PP) Method

A straightforward way to estimate the modal parameters of a structure subjected to ambient excitations, i.e., traffic, wind, micro-earthquakes, is the so-called PP method. This method is named after the key step of the method that the identification of the eigenfrequencies as the peaks of a spectrum plot. Under the conditions of well-separated eigenfrequencies, the natural frequency of a structure can be evaluated by picking the peak of the response spectrum. The spectrum can be obtained by the fast Fourier transform (FFT) of a time series of response. Due to its simplicity in implementation, the method is widely used for modal identification in many studies [69,70]. 

#### 3.1.2. The Random Decrement Technique (RDT) Method

Despite the advantages of PP method, i.e., straightforward and simplicity, it is hard to be used for damping and mode shape identification. The RDT was introduced by Cole in 1960s and 1970s [71,72]. Since then, it has become one of the most popular methods used for modal identifications. The output signals obtained from a SHM system can be extracted by using the RDT method that averages time series $x(t_{n})$ when a trigger condition is fulfilled (a level crossing trigger of trigger level a is supposed). The result of the averaging process is called a random decrement signature $D_{XX}(\tau )$. 

(1) $$D_{XX}(\tau )=\frac{1}{N}\sum_{n=1}^{N}x(t_{n}+\tau )|x(t_{n})=a$$

**Figure 9:**
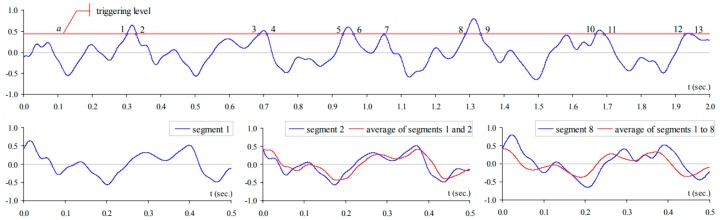
Basic concept of the RDT after [74].

At each time instant, the response observed from a SHM system includes three components: the response to an initial displacement, the response to an initial velocity, and the response to random input loads during the time span between the initial state and the regraded time instant [73]. By averaging a large number of time segments of the response, the random part of the response will disappear and the response of the system corresponding to the initial condition defined by the trigger is remained, containing the behavior of the system. The basic concept of the RDT is also interpreted in Figure 9. 

The RDT has been widely used in civil structures in damping estimation, frequency and mode shape identification [75,76,77]. The RDT has also been extensively developed in line with time domain identification methods, such as the Ibrahim time domain method, the eigensystem method. Details about the combined application can be found in a previous study [74]. 

#### 3.1.3. The Frequency Domain Decomposition (FDD) Method

The FDD technique that has been extensively used for modal identification was proposed by Brincker et al. [78]. The efficiency of the technique has been validated and widely used for modal identification of output systems [79,80]. The technique is briefly recalled below.

The first step to obtain the PSD matrix of ambient responses, $S_{yy}(j\omega )$. The output PSD is then decomposed at discrete frequencies $\omega =\omega_{i}$ by using the singular value decomposition, expressed as

(2) $$S_{yy}(j\omega_{i})=U_{i}G_{i}U_{i}^{H}$$

That the matrix $U_{i}=[U_{i1},U_{i2},\cdots ,U_{in}]$ is a matrix of the singular vectors; $G_{i}$ is the diagonal matrix of the scalar singular values. Close to a peak corresponding to the k-th mode in the spectrum, only a possible close mode is dominant and the PSD matrix approximates to a rank-one matrix is decomposed as

(3) $$S_{yy}(j\omega_{i})=U_{i1}G_{i1}U_{i1}^{H}\begin{array}{cc} & \omega_{i} \end{array}\to \omega_{k}$$

Therefore, the corresponding first singular value is the auto-spectral density function of a single degree of freedom (SDOF) system, and the first singular value is an estimate of the mode shape. From the density function obtained around the peak of the PSD, the natural frequency, damping ratio, and mode shape can be conveniently obtained. In order to get the PSD function of a SDOF system, the modal assurance criterion (MAC) is adopted. Details can be found in previous studies [78].

By using the above techniques, the modal parameters of an arch bridge can be identified and the identified results can be utilized for further analysis.

### 3.2. Methodologies on Signal Processing

Many mathematic models have been developed to process signals observed from a SHM system in association with time domain, frequency domain, or time-frequency domain. The models mainly include statistical time series (STS) models, and Kalman Filter (KF), fast fourier transform (FFT), short-time FFT (SFFT), wavelet transform (WT), S-transform (ST), fast ST (FST), Hilbert transform (HT), Hilbert-Huang transform (HHT), Multiple Signal Classification (MUSIC), Blind Source Separation (BSS), etc. By using the methods, the features of a structure, i.e., frequency, damping, damage, can be extracted based on vibration data obtained from a SHM system. For the purpose of convenient selection and use of the mentioned models above, the advantages and disadvantages of the models are summarized in Table 2. 

### 3.3. Damage Identification

Damage identification is one of the most important aspects for health evaluation of a structure based on the data obtained from a SHM system. Researchers have made their great efforts to explore related methods and theories for damage identification of structures. The modal-based damage detection is one of traditional methods that are utilized for damage identification. The basic idea of the method is that the modal parameters (i.e., frequency, damping ratio, mode shape, or their combinations) are functions of the physical properties of a structure and changes caused by damage will therefore lead to changes in modal parameters. Usually, damage will decrease the mass and stiffness of a structure. The modal-based damage detection method mainly includes several categories: modal shape change method, modal shape curve method, sensitivity-based update method, stiffness change method, frequency-response method, and combined modal parameter method [82]. Among them, mode shape method is often used for damage detection as it is less affected by environment [83]. The modal assurance criterion (MAC) has been developed, which can be used for measuring mode shape changes over the entire span of a structure [84]. Kim et al. [85] later updated the MAC and developed the coordinate modal assurance criterion (COMAC) that can monitor modal node displacement to detect and localize damage. Most recently, Magalhaes and Caetano [25] identified the damage of an arch bridge based on vibration data observed from a SHM system by using an automated operational modal analysis. Liu et al. [86] proposed a damage detection model that is related to the curvature of a concrete bridge. The efficiency of the model has been validated by experiment. Materazzi and Ubertini [29] have investigated the damage detection of long-span suspension bridges by using modal eigenproperties. Hanif et al. [87] has proposed a simulation based damage detection method based on linear and nonlinear analysis. The method has been validated through comparing the result with that in previous studies. 

Despite the recent progress in models and theories of damage detection, there still exist many challenges [84]: (1) environmental and operational variability, which will affect the stiffness and mass in a nonlinear manner and thus affect modal properties; (2) separating environmental variation from damage, which is a big challenge even though there are such techniques, i.e., the Least Trimmed Squares (LTS) regression algorithm and the Minimum Covariance Determinant (MCD) estimator [88]; (3) errors in non-modal based damage detection; (4) Damage localization. 

### 3.4. Some Results of a Long-Span Arch Bridge

**Figure 10:**
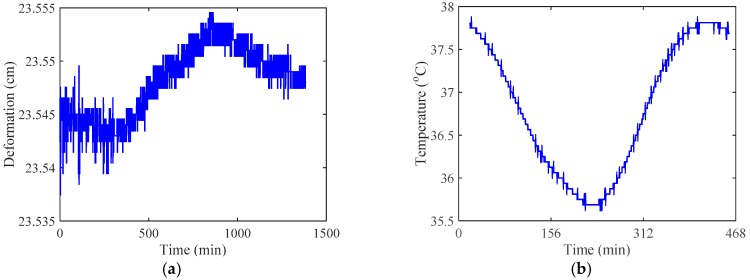

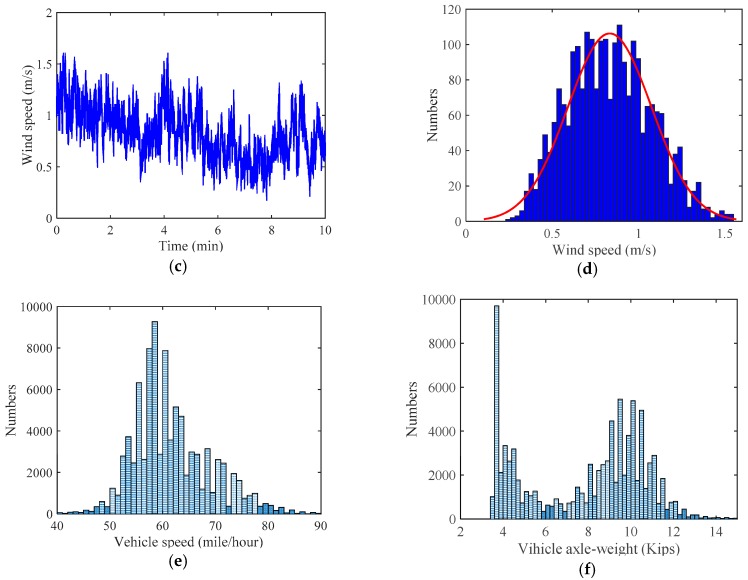
Monitoring interface: (**a**) observed deformation; (**b**) temperature; (**c**) time-history wind speed; (**d**) statistical analysis of wind speed; (**e**)statistical analysis of vehicle speed; (**f**) statistical analysis of vehicle axle-weight.

Section 2.5 introduces the deployment of a SHM system for a long-span arch bridge (the Jiubao Bridge in China). Through the system, some data about deformation, temperature, wind speed, vehicle, etc. was observed. Taking the 1-day observed data for an example, the results on observed deformation, temperature, time-history wind speed, statistical analysis of wind speed, statistical analysis of vehicle speed, statistical analysis of vehicle axle-weight and vibration mode are depicted in Figure 10. 

In Figure 10a,b, the start point ‘0’ is corresponding to zero o’clock. Figure 10a shows that the deformation of the bridge increases with time until a peak occur at the time around 800 min past zero o’clock (around 15:00 o’clock). The deformation is mainly affected by vehicle flowrate and the result therefore suggests that the vehicle flowrate is maximum around 15:00 o’clock. Figure 10b indicates that the lowest temperature occurs at the time around 230 min past zero o’clock (around 4:00 o’clock), and the maximum temperature is close to 38 °C that is identical to the temperature observed by the China Meteorological Administration. Figure 10c,d show that the wind speed is around 1.25 m/s and it substantially obeys to the normal distribution. At high wind speeds (i.e., typhoon), the measured wind speed would be of non-stationary feature and it does not obey the normal distribution any more [89]. Figure 10e,f are obtained based on one-month vehicle data observed from the SHM of the bridge. Figure 10e,f suggest that the vehicle speed substantially obeys the normal distribution whereas the vehicle axle-weight obeys the bimodal distribution. This is identical to a previous study [56]. 

**Figure 11:**
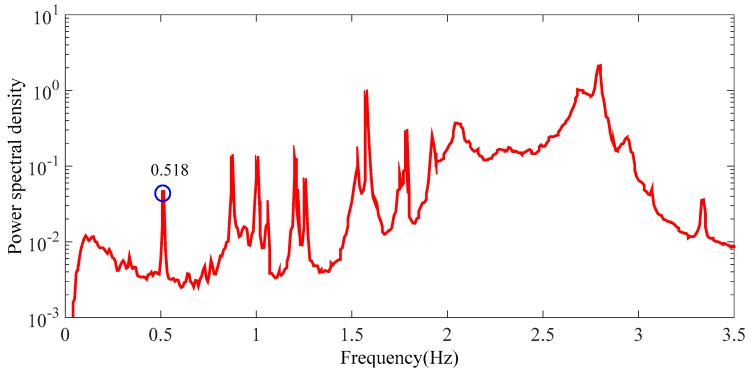
Average power spectral density of all vibration-monitoring points after [90].

The time-history response (acceleration) of the bridge measured from the SHM system can be found in a previous study [90]. By using proper analytical method (i.e., the PP method) illustrated above, some features (i.e., frequency) of the bridge are obtained. The power spectral density of all vibration-monitoring points is depicted in Figure 11. Figure 11 indicates that the fundamental frequency of the bridge measured by the SHM system is around 0.5 Hz that is identical to that calculated by using a 3-D finite element analysis (FEA) method (0.518 Hz) reported in the previous study [90]. The above results suggest that the deployment of the SHM of the long-span arch bridge is reliable.

## 4. Applications of SHM Systems for Arch Bridges

In the 1950s, the United States and some other developed countries established a substantial number of codes and standards for bridge inspection and retrofit. However, due to technology limitations at that time, artificial method was the only way for bridge testing, which leads to significant discrepancies between field measurements and real conditions. With the constant improvement of bridge health monitoring and diagnostics system in 1980s, the first batch of health monitoring system was established and applied to multiple bridges, e.g., the Foyle Bridge in the United Kingdom [91], the Skarnsundet cable-stayed bridge in Norway [92], and the Sunshine Skyway Bridge in the United States [93]. Since then, SHM systems have been implemented in many long-span bridges in China, Japan, America, Europe. Most of them equipped with SHM systems are cable-stayed bridges, and the number of arch bridges is relatively small (around 14%). Table 3 presents 10 typical long-span arch bridges in the world that have been equipped with SHM systems. 

From the above illustration, SHM systems for long span cable-stayed bridges and suspension bridges have been widely developed all over the world, but the systems on long-span arch bridges are relatively limited. The study is anticipated to advance our understanding on deployment and study of SHM of long-span arch bridges. 

## 5. Challenges and Future Trends

With the development of society, many progresses have been archived in the area of SHM of civil structures. However, there still exist many challenges. Though many studies [49,96] have summarized and recognized the challenges and future trends of SHM for civil structures, the field of SHM develops rapidly, and visions should be up-to-date in time. The challenges and future trends are summarized below.

• The main challenge for a SHM system is to obtain the exact damaged structural model. One the important ways to solve the problem is to develop advanced sensors. The stability, durability, and accuracy of sensors are of great important in developing a reliable SHM system. With the development of smart materials, high quality sensors are anticipated to be developed and utilized in SHM systems;
• Wireless sensing technologies or mobile wireless sensing technologies with high-frequency range and high accuracy should be developed;
• Data-driven science and technologies, including highly efficient data acquisition, data storage technologies, data management technologies, data processing technologies, data analysis and modeling technologies are important issues. New technologies, i.e., big data and cloud technologies, artificial intelligence, deep learning, are anticipated to be used to solve the issues;
• Identify damage accurately and quantitatively. The challenges of damage identification include (1) environmental and operational variability, which will affect the stiffness and mass in a nonlinear manner and thus affect modal properties; (2) separating environmental variation from damage, which is a big challenge even though there are such techniques; (3) errors in non-modal based damage detection; (4) Damage localization. New science and technologies are anticipated to be developed to solve the challenges;
• Other challenges, i.e., long-term condition assessment, life-cycle ultimate capacity prediction.

In a future SHM system, data is anticipated to be accurately collected, transmitted, stored, and analyzed in a short time by a SHM system where it is in conjunction with a structural control system. In a word, a future SHM system must realize the goal, evaluating the serviceability, safety, and sustainability of structures effectively.

## Figures and Tables

**Table 1:** Sensors often utilized in a sensory sub-system.

Monitoring Item	Variables	Sensors	Examples
**Loads and Environmental actions**	Vehicle load	Weigh-in-motion (WIM)	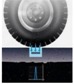
		Camera	/
	Wind load	Ultrasonic anemometer	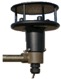
		Mechanical anemometer	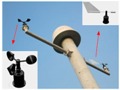
	Earthquake ground motion	Seismometer	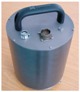
	Vessel collision	Accelerometer/Seismometer	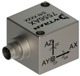
	Temperature and humidity	Temperature and humidity sensor	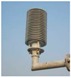
**Global Response**	Vibration	Accelerometer	/
	Displacement	Pressure transmitter sensor/GPS	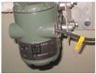
	Strain	Optical fiber Bragg grating (FPG) strain sensors	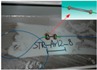
**Local Response**	Bearing displacement	Magnetostrictive displacement sensors	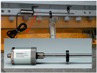
	Hanger rod/Cable force	Fiber brag grating test-force rings	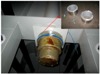

**Table 2:** Methods for signal processing after [81].

Methods	Advantages	Disadvantages
**STS**	Linear model; Ease of implementation	Sensitive to noise; Only used for linear systems
**KF**	Good signal-noise ratio; Good estimation of change in time	Time consuming; Requires parameter calibration; Limited convergence speed and tracking accuracy
**FFT**	Nonlinear model; Model linear and nonlinear systems; Ease of implementation; Simplicity	Not applicable for complex system; Requires calibration to find model order; Sensitive to noise; Only frequency domain representation
**MUSIC**	High resolution in frequency domain; Closely-spaced modes can be estimated	Time consuming
**SFFT**	Ease of implementation; Time-frequency domain representation; Simplicity	Requires large quantity of samples; Limited time-frequency resolution; Not applicable for nonlinear and transient signals
**WT**	Good time-frequency resolution; Good signal-noise ratio; A mother wavelet can be used for different application	Spectral leakage; Requires several levels of decomposition;Mother wavelet will affect the result; ‘End effect’ is significant
**ST**	Good time-frequency resolution; Spectrum can be localized in time domain	Time consuming; Requires calibration
**FST**	Time saving; Good time-frequency resolution; Spectrum can be localized in time domain	The application in SHM systems need exploring
**HHT**	Good time-frequency resolution; High signal-to-noise ratio; Adaptive method; Ease of implementation	Mode-mixing; Requires calibration
**BSS**	Good signal-noise ratio; Closely-spaced modes can be estimated; Good accuracy to separate frequency components	Require calibration Nonlinear and transient signals cannot be analyzed adequately

**Table 3:** Main arch bridges in the world equipped with SHM systems.

No.	Project Name	Location	Main Span (m)	Sensors [17,33]
1	Lupu bridge	Shanghai, China	550	(2)–(4), (9)
2	Banghwa Bridge	Seoul, Korea	540	(1)–(5), (8)
3	Sydney Harbour Bridge [24,94]	Sydney, Australia	503	(2)–(5)
4	Mingzhou Bridge	Zhejiang, China	450	(1)–(7), (10), (11)–(12)
5	Boguan Bridge	Liaoning, China	430	(1)–(4), (7)
7	Caiyuanba Bridge [95]	Chongqing, China	420	(2)–(5), (10)
8	Maocao Street Bridge	Hunan, China	368	(1)–(5), (7), (12)
9	Yonghe bridge	Guangxi, China	338	(1)–(5), (7), (12)
10	Dashengguan Yangtze River Bridge [26]	Jiangsu, China	336	(3)–(4), (6)

Note: (1) anemometer, (2) temperature sensor, (3) strain gauge, (4) accelerator, (5) displacement transducer, (6) velocimeter, (7) global positioning system, (8) tiltmeter, (9) level sensing station, (10) cable tension force, (11) ultrasonic wind speed and direction instrument, (12) video camera.
